# Relationship Between Peripheral Refraction in Different Retinal Regions and Myopia Development of Young Chinese People

**DOI:** 10.3389/fmed.2021.802706

**Published:** 2022-01-18

**Authors:** Xiangyue Zheng, Dejin Cheng, Xiaoli Lu, Xiaoyi Yu, Yuting Huang, Yujie Xia, Chuni Lin, Zhao Wang

**Affiliations:** ^1^Department of Ophthalmology, The First Affiliated Hospital of Guangzhou University of Chinese Medicine, Guangzhou, China; ^2^Department of Nephropathy, The First Affiliated Hospital of Guangzhou University of Chinese Medicine, Guangzhou, China; ^3^First Clinical Medical School, Guangzhou University of Chinese Medicine, Guangzhou, China

**Keywords:** peripheral refraction, myopia, multispectral refractive topography, ocular biometrics, retinal eccentricity

## Abstract

**Objective:**

To observe the associations between regional peripheral refraction and myopia development in young Chinese people.

**Methods:**

Two hundred and forty-one young adult subjects (21 emmetropes, 88 low myopes, 94 moderate myopes, and 38 high myopes) aged 18–28 years were included, and only the right eyes were tested. Eye biometrics were measured before pupil dilation using the Lenstar. Relative peripheral refractive errors (RPRE) were measured after pupil dilation using multispectral refractive topography (MRT), at nine retinal eccentricities: 0–5, 5–10, 10–15, 15–20, 20–25, 25–30, 30–35, 35–40, and 40–45 degrees.

**Results:**

In this study, RPRE increased with eccentricity, and it shows a growing trend with the increase of the degree of myopia among emmetropia, low myopia and moderate myopia groups, and RPRE varied with myopia severity at eccentricities between 20 and 35 degrees only. In addition, axial length (AL) and RPRE were positively correlated between 20 and 45 degrees, and AL was an independent risk factor for RPRE between 20 and 35 degrees.

**Conclusion:**

These findings indicate that the eccentricities between 20 and 35 degrees RPRE may be closely related to refractive development and eye growth in young Chinese people.

## Introduction

Peripheral hyperopic defocus has been an area of research interest in the pathogenesis of myopia in recent years and peripheral refraction is of great significance in the field of vision research.

Studies have shown that the human visual system can recognize signs of defocus and change its axial length, causing the retina to migrate toward the defocused image plane (1–5). Therefore, peripheral defocus, especially the relative hyperopic defocus, has an important impact on eye growth and refractive error progression (4–7). As the interest in peripheral defocus has grown, an almost constant stream of related research has emerged. However, few studies have been conducted on the refractive status at different retinal eccentricities and their association with myopia. The equipment for this type of study is traditional but its operation is complex. In this study, a new approach known as multispectral refractive topography (MRT) was used to measure the relative peripheral refractive errors (RPRE) at a range of eccentric retinal regions. This approach differs from traditional methods, such as the infrared autorefractor and wavefront aberration analyzer, by using a single (rather than multiple) target, and no interference from eye muscle contraction and adjustment changes during the process. In addition, MRT provides accurate measurement at a wide range of eccentricities from 0 to 45°, with good repeatability and accuracy. In this study, we used MRT to measure RPRE at different retinal regions in young Chinese people and explored associations between RPRE and myopia development in this group.

## Materials and Methods

### Study Population

**Figure 1:**
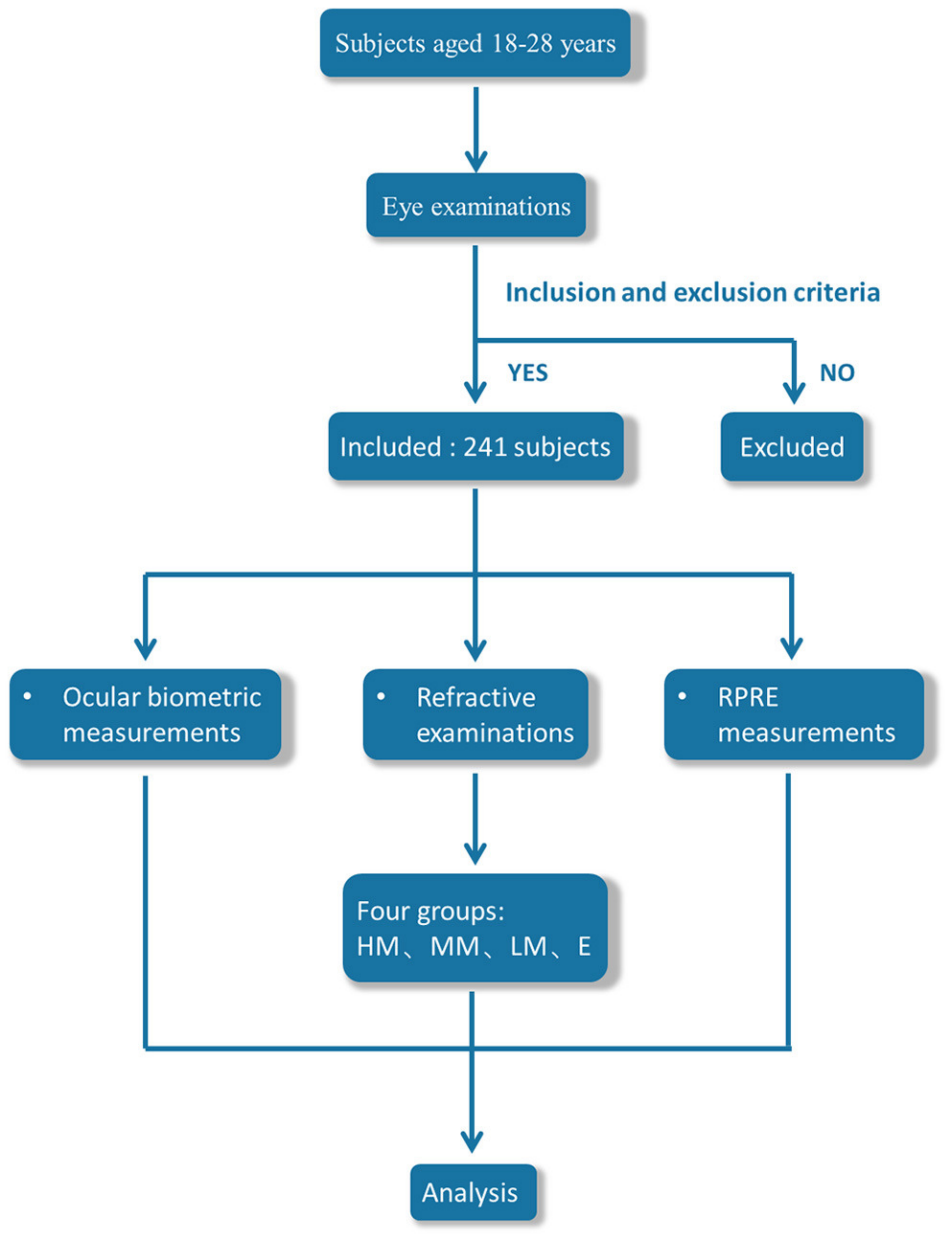
Flowchart of experimental design.

There were 241 patients (241 eyes; only right eyes were considered) with myopia or emmetropia aged 18–28 years, who were admitted to the Ophthalmology Department of the First Affiliated Hospital of Guangzhou University of Traditional Chinese Medicine, included in this study. The best-corrected visual acuity in the right eye was required to be at least 1 with no other ocular condition or disease, and subjects were excluded if they had a history of ocular surgery or of wearing contact lenses. Subjects were classified into four refractive groups according to central spherical equivalent (SE) refractive error: Emmetropia group (E, +0.5 to −0.5 D), Low Myopia group (LM, −0.50 to −3 D), Moderate Myopia group (MM, −3 to −6 D), and High Myopia group (HM, >-6 D) (Figure 1). The study was conducted in accordance with the tenets of the Declaration of Helsinki, and a written informed consent was obtained from all participants. Ethical approval was obtained from the Institutional Review Board of the First Affiliated Hospital of Guangzhou University of Traditional Chinese Medicine.

### Eye Examinations

Before the study, each subject underwent an eye examination to ensure a good ocular health and to determine their refractive status. Eye examinations were performed by trained ophthalmologists and optometrists.

Refraction was measured using an autorefractor (Topcon KR-800, Topcon Co. Tokyo, Japan) under cycloplegia, 30 min after two drops of the Compound Tropicamide eye drops (consisting of 25 mg tropicamide and 25 mg phenylephrine; Tianjin Kingyork Group Hebei Univision Pharmaceutical Company Limited). Refractive error was recorded as Sphere (S), Cylinder (C), and Axis then converted into vector components. The equation is SE = S + C/2, where SE value was used to categorize the eyes into four refractive groups as described above.

All ocular biometric measurements were made using the Lenstar LS900 optical biometer (Lenstar LS 900; Haag Streit AG, Koeniz, Switzerland) before pupil dilation, and included axial length (AL), central corneal thickness (CCT), anterior chamber depth (AD), lens thickness (LT), keratometry (K1, K2), and astigmatism (AST = K2–K1). A total of 3 measurements of each parameter were made and the average was obtained.

**Figure 2:**
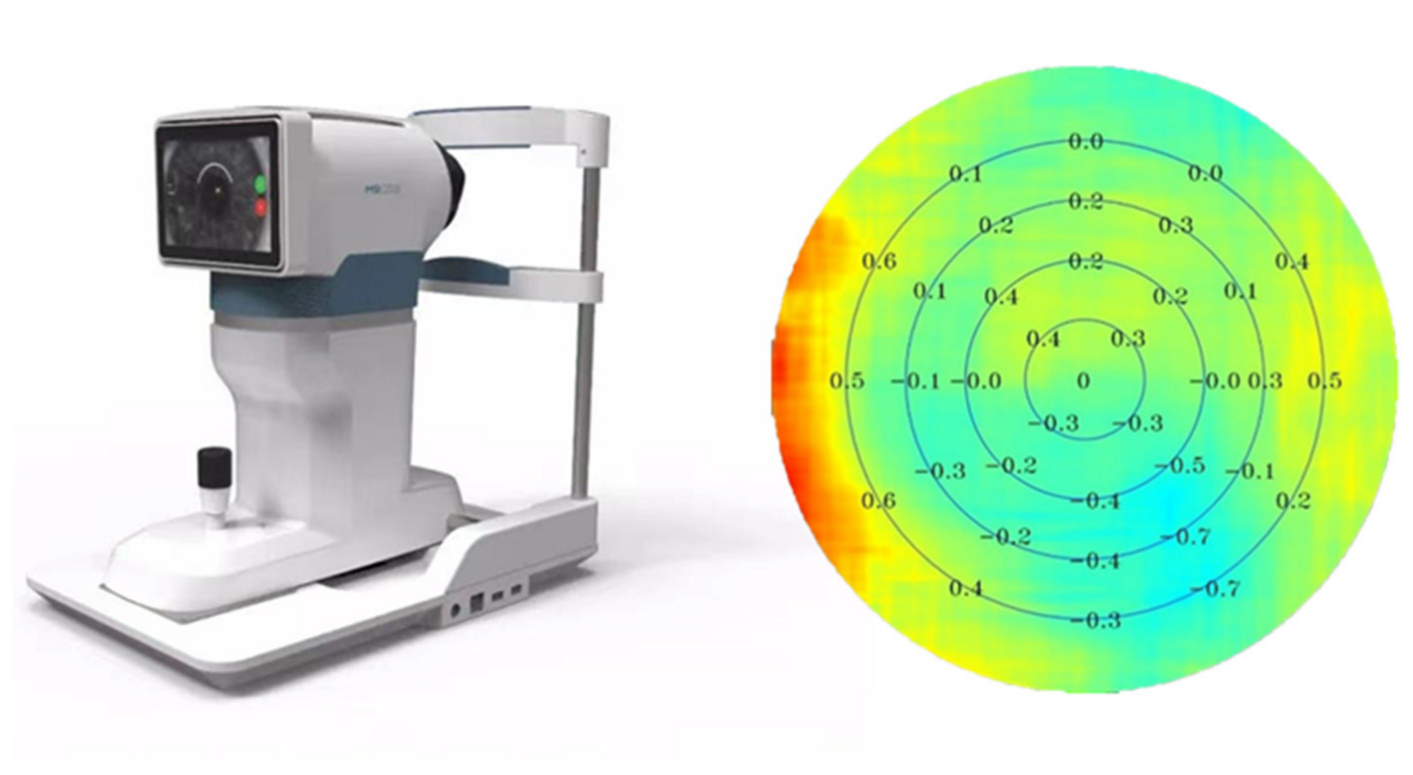
Multispectral refraction topography and the report.

Multispectral Refraction Topography (MRT, MSI C2000, ShengDa TongZe, ShenZhen, China) (Figure 2) was used to measure the RPREs after complete mydriasis. The RPRE was calculated at each eccentricity as the difference between SE at the central and eccentric locations (8); a hyperopic RPRE is represented in the results by positive values, while a myopic RPRE is represented by negative values. Peripheral refractions were determined at eccentricities of 0–5, 5–10, 10–15, 15–20, 20–25, 25–30, 30–35, 35–40, and 40–45 degrees. During this process, the patients were required to fixate a green target straight ahead. All measurements were obtained in a single session, and monocular photography was completed in 5–10 s without the need for eye rotation to look at multiple targets.

### Statistical Analysis

The SPSS 20.0 statistical software (Chicago, IL) was used for statistical analysis. Normally distributed data were expressed as $\bar{\text{X}}\pm$S, comparisons between groups were analyzed using one-way ANOVA, and pairwise comparisons were made using Tukey's method. Non-normal data were expressed as median and quartiles, and a Kruskal-Wallis *H*-test was used for statistical analysis. To study the relationship between AL, CCT, AD, LT, K1, AST, and RPRE, Spearman's correlation analysis and multiple linear regression analysis were used. The level of significance was set at 5%.

## Results

### Descriptive Characteristics

Among the 241 patients, there were 97 men and 144 women, aged 18–28 years old. Of the 241 eyes, 21 were emmetropic, 88 had low myopia, 94 had moderate myopia, and 38 had high myopia. Among the four refractive groups, there were no statistically significant differences in gender or age, and the mean values of SE, AL, CCT, AD, LT, AST, and K1 are all presented in Table 1.

**Table 1:** Biometric data of four refractive groups, M (Q25, Q75) or $\bar{X}$ ± S.

	**E**	**LM**	**MM**	**HM**	***P***
Number	21	88	94	38	
Gender (male/female)	10/11	37/51	35/59	15/23	0.812
Age (years)	22 (18, 24)	21.5 (18, 25)	22 (18, 24)	22.5 (22, 24)	0.315
SE (D)	−0.280 ± 0.213	−1.771 ± 0.667	−4.197 ± 0.973	−8.109 ± 2.024	0.000
AL (mm)	23.547 ± 0.742	24.236 ± 0.737	25.212 ± 0.845	26.868 ± 1.000	0.000
CCT (μm)	552.421 ± 30.174	554.268 ± 33.690	554.598 ± 30.366	543.111 ± 22.299	0.258
AD (mm)	3.018 ± 0.198	3.129 ± 0.446	3.156 ± 0.249	3.207 ± 0.206	0.228
LT (mm)	3.682 ± 0.332	3.412 ± 0.456	3.484 ± 0.229	3.587 ± 0.241	0.005
AST (D)	0.935 ± 0.584	1.080 ± 0.794	1.286 ± 0.863	1.652 ± 0.793	0.002
K1 (D)	42.944 ± 1.472	42.921 ± 1.438	42.801 ± 1.349	42.915 ± 1.317	0.940

### RPRE at Different Eccentricities Among Refractive Groups

**Figure 3:**
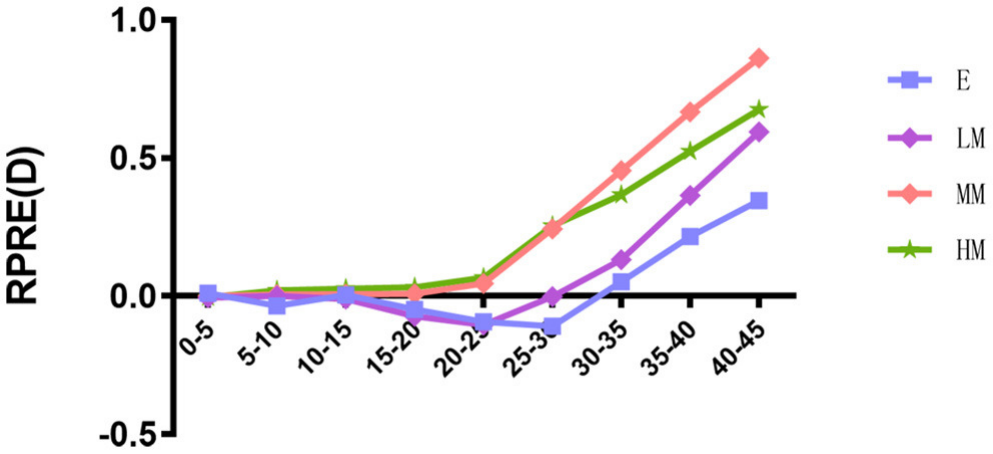
Trend of relative peripheral refractive errors (RPRE) in different eccentricities of four refractive groups.

The RPRE increased with increasing eccentricity. Patients with high and moderate myopia had relative hyperopia at all eccentricities, whereas patients with low myopia and emmetropia had relative hyperopia only beyond 30 and 35° eccentricities, respectively. At all eccentricities apart from those between 30 and 45°, the RPRE increased with myopia severity. At the higher eccentricities (30–45°), the converse was true, with RPRE reduced at higher eccentricity (Figure 3).

### RPRE Between Refractive Groups

**Figure 4:**
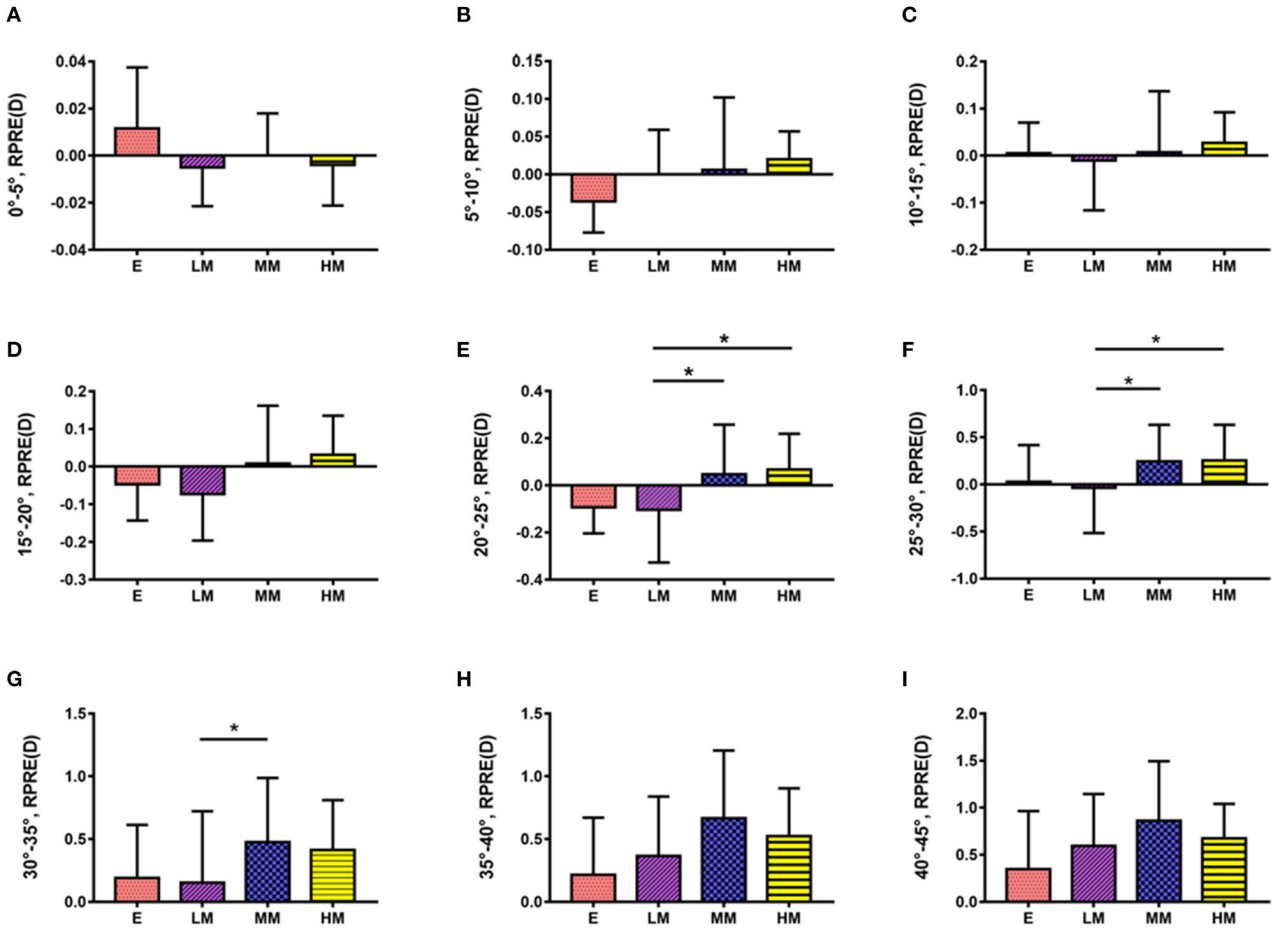
Comparison of RPRE among four refractive groups in eccentricities between 0 and 5°, 5 and 10°, 10 and 15°, 15 and 20°, 20 and 25°, 25 and 30°, 30 and 35°, 35 and 40°, and 40 and 45°. *p < 0.05.

The study showed no difference in RPRE among the four refractive groups at eccentricities between 0 and 20°, 35 and 45°(*P* > 0.05; Figures 4A–D,H,I).

At eccentricities between 20 and 25°, RPRE values were non-normally distributed, and the RPREs in the four refractive groups (E, LM, MM, and HM) were −0.093D (−0.204–0.116), −0.105D (−0.328–0.068), 0.046D (−0.127–0.258), and 0.067D (−0.038–0.219), respectively. The RPRE of the LM group was less than that of the MM and HM groups, respectively, and the differences were statistically significant (*P* < 0.05; Figure 4E).

At eccentricities between 25 and 30°, RPRE values were normally distributed, and the RPREs of the four refractive groups (E, LM, MM, and HM) were 0.026 D ± 0.392, −0.037 D ± 0.479, 0.244 D ± 0.388, and 0.255D ± 0.378, respectively. The RPRE of the LM group was less than that of the MM and HM groups, respectively, and the differences were statistically significant (*P* < 0.05; Figure 4F).

At eccentricities between 30 and 35°, RPREs were normally distributed, and the RPREs of the four refractive groups (E, LM, MM, and HM) were 0.189 D ± 0.424, 0.152 D ± 0.570, 0.477 D ± 0.511, and 0.413 D ± 0.398, respectively, and the RPRE was significantly different between the LM and MM groups (*P* < 0.05; Figure 4G).

### Relationship Between RPRE at Different Eccentricities and Ocular Biological Parameters

Spearman's correlation analysis showed a positive correlation between RPRE and AL at eccentricities between 20 and 45° (Table 2). Multiple linear regression analysis showed no significant relationship between the RPRE at eccentricities between 0 and 20, 40, and 45° and the ocular biological parameters (*P* > 0.05), while at eccentricities between 20 and 35°, AL was an independent risk factor for RPRE (*P* < 0.05; Tables 3–5), and at eccentricities between 35 and 40° CCT was an independent risk factor for RPRE (*P* < 0.05; Table 6).

**Table 2:** Correlation between relative peripheral refractive errors (RPRE) and axial length (AL) at different eccentricities.

	**0–5°**	**5–10°**	**10–15°**	**15–20°**	**20–25°**	**25–30°**	**30–35°**	**35–40°**	**40–45°**
r	−0.042	0.054	0.031	0.166	0.322	0.347	0.285	0.208	0.177
*P*	0.625	0.527	0.721	0.052	<0.001^*^	<0.001^*^	0.001^*^	0.015^*^	0.039^*^

* *P < 0.05 means RPRE has a significant correlation with AL*.

**Table 3:** Relationship between RPRE and ocular biological parameters at 20–25° eccentricity.

**Variates**		**OR (95% CI)**	***P***
AL	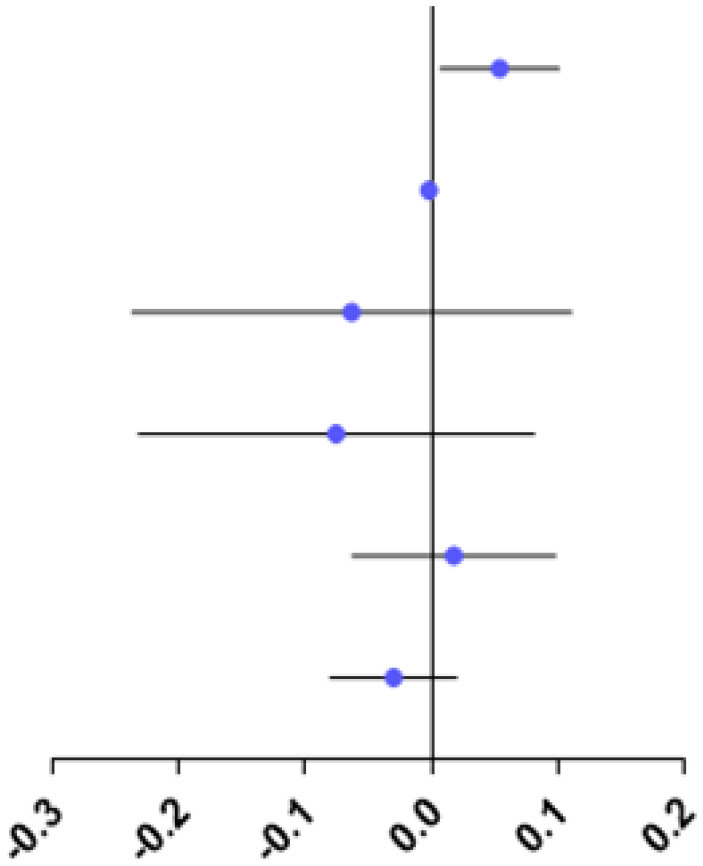	0.054 (0.007–0.101)	0.025^*^
CCT		−0.002 (−0.004–0.000)	0.071
AD		−0.063 (−0.237–0.111)	0.474
LT		−0.075 (−0.232–0.081)	0.342
AST		0.018 (−0.063–0.098)	0.663
K1		−0.030 (−0.080–0.020)	0.239

* *P < 0.05, AL is an independent risk factor for RPRE*.

**Table 4:** Relationship between RPRE and ocular biological parameters at 25–30° eccentricity.

**Variates**		**OR (95% CI)**	***P***
AL	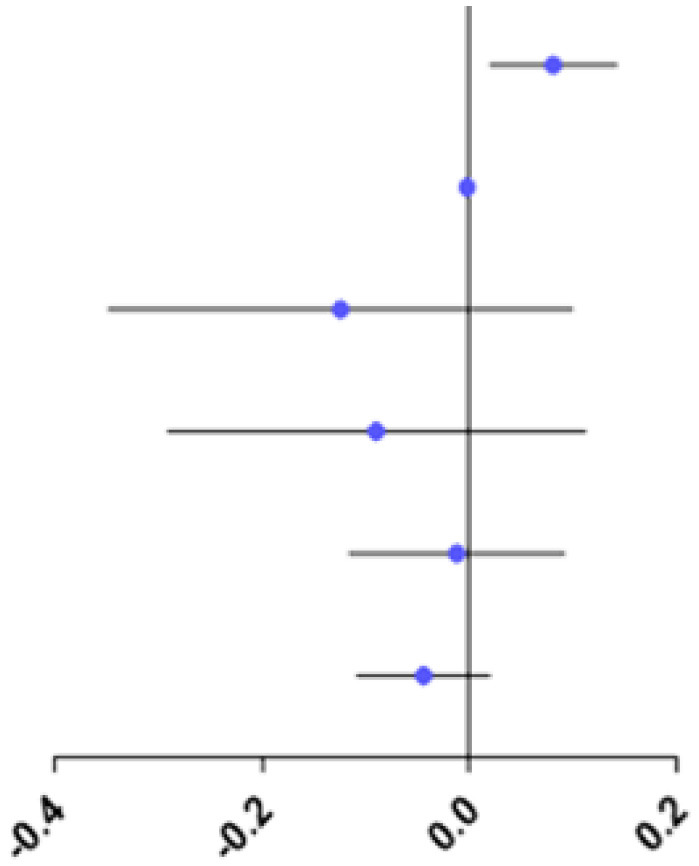	0.081 (0.020–0.142)	0.009^*^
CCT		−0.002 (−0.004–0.000)	0.098
AD		−0.124 (−0.348–0.100)	0.274
LT		−0.090 (−0.291–0.112)	0.380
AST		−0.012 (−0.116–0.092)	0.821
K1		−0.044 (−0.109–0.020)	0.177

* *P < 0.05, AL is an independent risk factor for RPRE*.

**Table 5:** Relationship between RPRE and ocular biological parameters at 30–35° eccentricity.

**Variates**		**OR (95% CI)**	**P**
AL	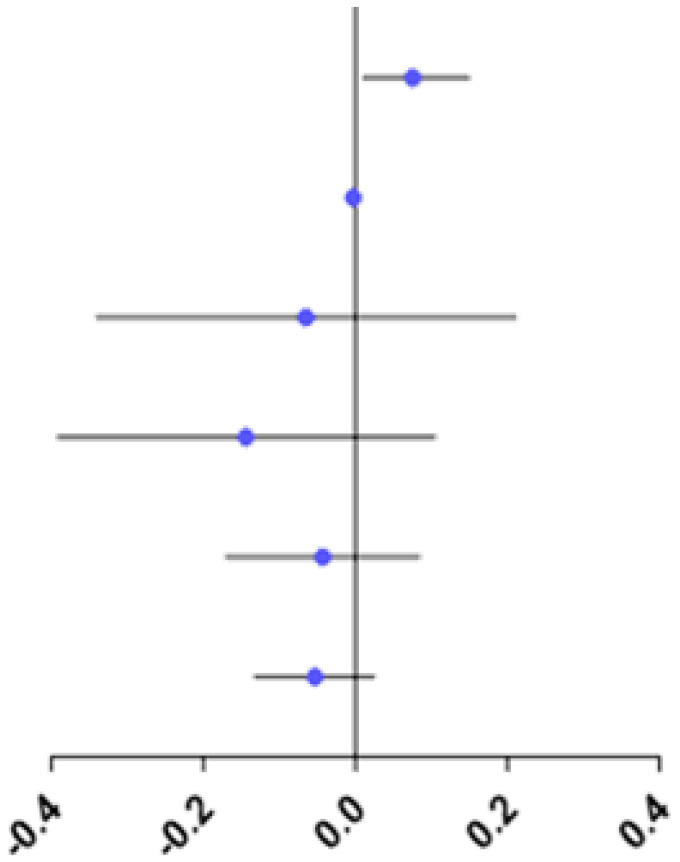	0.075 (0.009–0.150)	0.049^*^
CCT		−0.003 (−0.006–0.000)	0.058
AD		−0.065 (−0.341–0.212)	0.644
LT		−0.144 (−0.392–0.105)	0.255
AST		−0.043 (−0.171–0.085)	0.511
K1		−0.053 (−0.133–0.026)	0.188

* *P <0.05, AL is an independent risk factor for RPRE*.

**Table 6:** Relationship between RPRE and ocular biological parameters at 35–40° eccentricity.

**Variates**		**OR (95% CI)**	***P***
AL	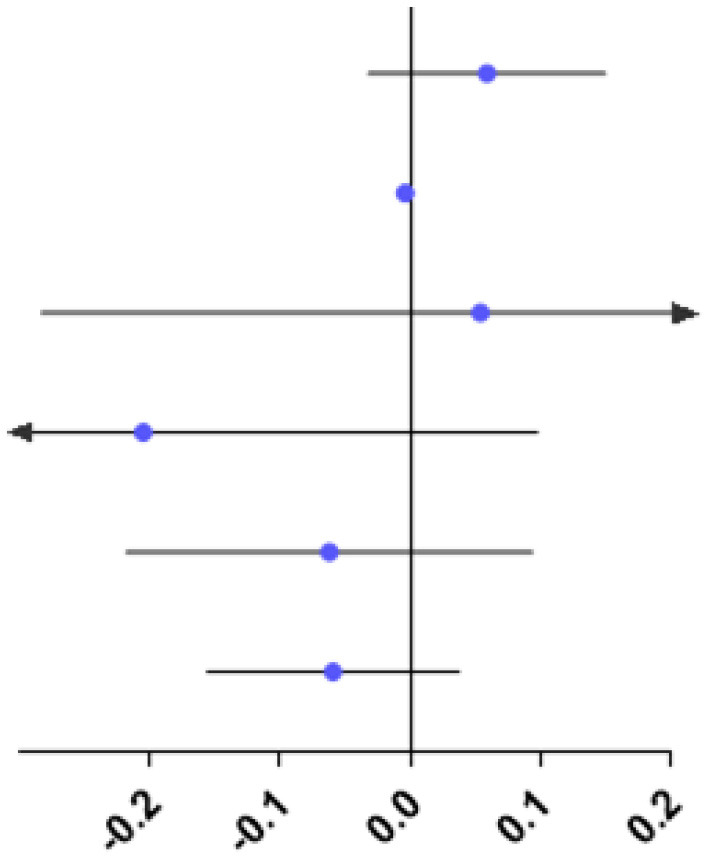	0.059 (−0.032–0.150)	0.203
CCT		−0.004 (−0.007–0.001)	0.045^*^
AD		0.054 (−0.283–0.391)	0.752
LT		−0.205 (−0.508–0.098)	0.184
AST		−0.062 (−0.218–0.094)	0.430
K1		−0.059 (−0.156–0.038)	0.232

* *P <0.05, CCT is an independent risk factor for RPRE*.

## Discussion

In this study, patients with high and moderate myopia had relative hyperopia at all eccentricities, whereas patients with low myopia and emmetropia had relative hyperopia only beyond 30 and 35° eccentricity, respectively. Similarly, Sng et al. (9) found that the eyes with moderate and high myopia showed peripheral relative hyperopia at all eccentricities, while those with low myopia showed peripheral relative hyperopia only at beyond 30° eccentricity. Mutti et al. (7) found that peripheral hyperopic defocus in children appears 2 years before the onset of myopia, indicating that it may also appear in emmetropia, consistent with our results.

Patients with different refractive degrees have different degrees of peripheral hyperopia defocus. Many studies have found that myopic eyes have greater RPRE than emmetropic or hyperopic eyes (10–12). Chen et al. (13) compared the RPRE in patients with different refractive errors and found that the hyperopic shift was greater in MM than in LM, but was similar in E and LM. Shen et al. (14) compared the RPRE in horizontal, vertical, and two diagonal meridians, and found greater hyperopic shift in HM than MM group on the horizontal and on the two diagonal meridians, while on the vertical meridian, hyperopic shift was greater toward the inferior visual field than toward the superior visual field in the MM and HM groups. The results of the present study are consistent with those of the above studies. Furthermore, we also found that beyond the 30° eccentricity, the RPRE in HM is lower than in MM group, and this finding needs to be confirmed since there are few related studies. In addition, the difference of the conclusion may also be related to the small number of patients with high myopia, or to the difference in measurement methods between different studies.

The relationship between peripheral refractive error and myopia remains controversial. Some studies show that peripheral hyperopic defocus is not related to the development of axial myopia (15), but the weight of evidence suggests that the two are associated (5–7). Knowledge about this association is lacking. Using a global flash multifocal electroretinogram (mfERG) under defocused conditions, Ho et al. (16) found that the different regions of the retina vary in their sensitivity to optical defocus and have different modifications in response to it. In a study on chicks, El-Nimri et al. (17) used bifocal lenses with different effective diameters to cause a range of levels of peripheral defocus and found that the influence of peripheral defocus on the refractive and axial length changes is varied across regions. The above findings, together with those of the present study, indicate that the peripheral retina may have major and secondary influences on eye growth and refractive development. Similar to the above research results, we found that patients with different degrees of myopia have significant differences in RPRE at eccentricities between 20 and 35°, the axial length was positively correlated with RPRE, and we infer that defocus in this region may be an important factor affecting the onset and development of myopia.

At present, the mechanism by which the peripheral defocus affects axial length growth requires further study. It may be related to retinal control of growth of the underlying sclera (18). In this case, form deprivation in the peripheral retinal may promote local growth of the eye, including the axial length (11). Alternatively, signals from the relatively large number of neurons in the peripheral retina may inhibit those from the central retina, thus, directly regulating the eye growth and refractive development (10, 19–21). In addition, some studies have shown that bipolar cells and amacrine nerve cells detect defocus and are more sensitive to hyperopic than myopic defocus signals (22). These findings infer that the difference in sensitivity and distribution of these neurons and cells may be important factors in the varied retinal response to peripheral defocus in different regions, with varied effects on eye growth.

Numerous studies have shown that peripheral hyperopic defocus is an important trigger for the development of axial myopia (5, 6, 13). Conversely, some have argued that relative peripheral hyperopia in the relatively long myopic eye may be a result of eye growth (10). The present cross-sectional study could not determine whether peripheral defocus is the cause or effect of axial myopia, but we found a significant positive correlation between AL and RPRE, at eccentricities between 20 and 45°, and that AL is an independent risk factor for RPRE at eccentricities between 20 and 35°. Previous studies have also shown significant differences in peripheral refractive error between different types of myopia. Compared with refractive myopia, the peripheral defocus is higher than that of axial myopia (23), and AL is positively correlated with the refractive error of the peripheral hyperopia (9).

Our study showed that the RPRE of patients with different severities of myopia are significantly different mainly at eccentricities between 20 and 35°, and in this region, a significant positive correlation was found between RPRE and AL. Therefore, we infer that the refractive state of the peripheral retina between eccentricities of 20 and 35° might closely be related to the development of myopia.

## Limitations

The MRT instrument used in the present study is new and has not been widely used, hence, there is little prior research using this approach and research is needed to confirm the conclusions. The design of the cross-sectional study prevents investigation of whether the peripheral defocus is the cause or effect of axial myopia. We could only conclude that RPRE at eccentricities between 20 and 35 degrees may be closely related to the axial myopia in young Chinese people. In addition, this study did not consider some of the factors in addition to RPRE which may influence the eye development. Finally, the sample size of the groups varies, E and HM groups being particularly small, and this may also affect the accuracy of conclusions.

## Data Availability Statement

The raw data supporting the conclusions of this article will be made available by the authors, without undue reservation.

## Ethics Statement

The studies involving human participants were reviewed and approved by Institutional Review Board of the First Affiliated Hospital of Guangzhou University of Traditional Chinese Medicine. The patients/participants provided their written informed consent to participate in this study. Written informed consent was obtained from the individual(s) for the publication of any potentially identifiable images or data included in this article.

## Author Contributions

XZ and XY contributed to design, conduct of the study, reviewed, and finally approved the manuscript. XL, YH, YX, CL, and ZW collected data for the study. XY supervised the process. DC and XZ contributed to data analysis. XZ, DC, and XL prepared the manuscript. All authors contributed to the article and approved the submitted version.

## Funding

This study was supported by Innovation and strong academy project of the First Affiliated Hospital of Guangzhou University of Chinese Medicine (2019ZD03).

## Conflict of Interest

The authors declare that the research was conducted in the absence of any commercial or financial relationships that could be construed as a potential conflict of interest.

## Publisher's Note

All claims expressed in this article are solely those of the authors and do not necessarily represent those of their affiliated organizations, or those of the publisher, the editors and the reviewers. Any product that may be evaluated in this article, or claim that may be made by its manufacturer, is not guaranteed or endorsed by the publisher.

## Abbreviations

AD: Anterior chamber depth
AL: Axial length
AST: Astigmatism
C: Cylinder
CCT: Central corneal thickness
E: Emmetropia
HM: High Myopia group
K: Keratometry
LM: Low Myopia group
LT: Lens thickness
mfERG: Multifocal electroretinogram
MM: Moderate Myopia group
MRT: Multispectral refractive topography
RPRE: Relative peripheral refractive errors
S: Sphere
SE: Spherical equivalent.

## References

[1] Chiang ST Phillips JR Backhouse S. Effect of retinal image defocus on the thickness of the human choroid. Ophthalmic Physiol Opt. (2015) 35:405–13. 10.1111/opo.12218 26010292

[2] Delshad S Collins MJ Read SA Vincent SJ. The time course of the onset and recovery of axial length changes in response to imposed defocus. Sci Rep. (2020) 10:8322. 10.1038/s41598-020-65151-5 32433541 PMC7239843

[3] Chakraborty R Read SA Collins MJ. Hyperopic defocus and diurnal changes in human choroid and axial length. Optom Vis Sci. (2013) 90:1187–98. 10.1097/OPX.0000000000000035 24061153

[4] Smith EL III Hung LF Huang J. Relative peripheral hyperopic defocus alters central refractive development in infant monkeys. Vision Res. (2009) 49:2386–92. 10.1016/j.visres.2009.07.011 19632261 PMC2745495

[5] Smith EL III Kee CS Ramamirtham R Qiao-Grider Y Hung LF. Peripheral vision can influence eye growth and refractive development in infant monkeys. Invest Ophthalmol Vis Sci. (2005) 46:3965–72. 10.1167/iovs.05-0445 16249469 PMC1762100

[6] Smith EL 3rd Ramamirtham R Qiao-Grider Y Hung LF Huang J Kee CS. Effects of foveal ablation on emmetropization and form-deprivation myopia. Invest Ophthalmol Vis Sci. (2007) 48:3914–22. 10.1167/iovs.06-1264 17724167 PMC2709928

[7] Mutti DO Hayes JR Mitchell GL Jones LA Moeschberger ML Cotter SA. Refractive error, axial length, and relative peripheral refractive error before and after the onset of myopia. Invest Ophthalmol Vis Sci. (2007) 48:2510–9. 10.1167/iovs.06-0562 17525178 PMC2657719

[8] Lin Z Martinez A Chen X Li L Sankaridurg P Holden BA. Peripheral defocus with single-vision spectacle lenses in myopic children. Optom Vis Sci. (2010) 87:4–9. 10.1097/OPX.0b013e3181c078f1 19826316

[9] Sng CC Lin XY Gazzard G Chang B Dirani M Chia A. Peripheral refraction and refractive error in Singapore Chinese children. Invest Ophthalmol Vis Sci. (2011) 52:1181–90. 10.1167/iovs.10-5601 20926827

[10] Mutti DO Sholtz RI Friedman NE Zadnik K. Peripheral refraction and ocular shape in children. Invest Ophthalmol Vis Sci. (2000) 41:1022–30. 10752937

[11] Seidemann A Schaeffel F Guirao A Lopez-Gil N Artal P. Peripheral refractive errors in myopic, emmetropic, and hyperopic young subjects. J Opt Soc Am A Opt Image Sci Vis. (2002) 19:2363–73. 10.1364/JOSAA.19.002363 12469730

[12] Atchison DA Pritchard N Schmid KL. Peripheral refraction along the horizontal and vertical visual fields in myopia. Vision Res. (2006) 46:1450–8. 10.1016/j.visres.2005.10.023 16356528

[13] Chen X Sankaridurg P Donovan L Lin Z Li L Martinez A. Characteristics of peripheral refractive errors of myopic and non-myopic Chinese eyes. Vision Res. (2010) 50:31–5. 10.1016/j.visres.2009.10.004 19825388

[14] Shen J Spors F Egan D Liu C. Peripheral refraction and image blur in four meridians in emmetropes and myopes. Clin Ophthalmol. (2018) 12:345–58. 10.2147/OPTH.S151288 29497275 PMC5822858

[15] Schippert R Schaeffel F. Peripheral defocus does not necessarily affect central refractive development. Vision Res. (2006) 46:3935–40. 10.1016/j.visres.2006.05.008 16806391

[16] Ho WC Wong OY Chan YC Wong SW Kee CS Chan HH. Sign-dependent changes in retinal electrical activity with positive and negative defocus in the human eye. Vision Res. (2012) 52:47–53. 10.1016/j.visres.2011.10.017 22100834

[17] El-Nimri NW Zhang H Wildsoet CF. The effect of part-time wear of 2-zone concentric bifocal spectacle lenses on refractive error development & eye growth in young chicks. Exp Eye Res. (2019) 180:184–91. 10.1016/j.exer.2018.12.010 30582914 PMC6398993

[18] Wallman J Gottlieb MD Rajaram V Fugate-Wentzek LA. Local retinal regions control local eye growth and myopia. Science. (1987) 237:73–7. 10.1126/science.3603011 3603011

[19] Schmid GF. Variability of retinal steepness at the posterior pole in children 7-15 years of age. Curr Eye Res. (2003) 27:61–8. 10.1076/ceyr.27.2.61.15454 12868010

[20] Logan NS Gilmartin B Wildsoet CF Dunne MC. Posterior retinal contour in adult human anisomyopia. Invest Ophthalmol Vis Sci. (2004) 45:2152–62. 10.1167/iovs.03-0875 15223789

[21] Wallman J Winawer J. Homeostasis of eye growth and the question of myopia. Neuron. (2004) 43:447–68. 10.1016/j.neuron.2004.08.008 15312645

[22] Zhong X Ge J Smith EL 3rd Stell WK. Image defocus modulates activity of bipolar and amacrine cells in macaque retina. Invest Ophthalmol Vis Sci. (2004) 45:2065–74. 10.1167/iovs.03-1046 15223778

[23] Bakaraju RC Ehrmann K Papas EB Ho A. Do peripheral refraction and aberration profiles vary with the type of myopia?–An illustration using a ray-tracing approach. J Optom. (2009) 2:29–38. 10.3921/joptom.2009.29

